# Chaptal's Letter to the Prefects, on Vaccination

**Published:** 1805-05-01

**Authors:** John Ring

**Affiliations:** New Street, Hanover Square


### *To the Editors of the Medical and Physical Journal.*

GENTLEMEN,

WHILE Vaccination, in this its native kingdom, is principally promoted by a few humble and obscure individuals, it is more justly appreciated in other countries. In France, it is cultivated with unremitting ardor; and all the energies of Government are called forth in support of the practice.

Its propagation is there encouraged by something more than barren patronage, and empty names. It is there considered as a national concern. The Minister of the Interior has instituted a Society for the Extermination of the Small-pox, over which he himself presides; and under his auspices, upwards of sixty thousand human beings have been secured from the ravages of the small-pox, within the space of three months.

I here subjoin a translation of the Plan of that Society; and cannot conclude without declaring, that I would rather be Chaptal at the head of his benevolent Institution, than Bonaparte in all his glory.

I am, &c.

*New Street, Hanover Square.* JOHN RING.

---

"MINISTER OF THE INTERIOR.

"Society for the Extermination of the Small-pox in France, "by Means of Vaccination.

"Letter of the Minister to the Prefects.

"*Paris, 14 Germinal, An. 12.*

"I invited you, Citizen Prefect, by my circular letter of last Prairial, to promote by all the means in your power Vaccine Inoculation; the immense advantages of which, for the increase of population, and the welfare of mankind, are so completely demonstrated.

"The Prefects in many departments are already impressed with a zeal worthy of the highest eulogiums, to fulfil in this respect the paternal views of Government; by establishing Committees of Vaccination, and organising the practice of this salutary art, in the different districts submitted to their care. It is to second their generous efforts, to strengthen them, and to regulate them by a general impulse, that I again call your attention to this important subject.

"The Central Committee of Vaccination, whose report has reflected so much light upon that novel species of practice, have expressed a wish, that a new society for propagating vaccine inoculation should be established; for the purpose of accomplishing the extermination of the small-pox in France,—an object of the highest concern; the practicability of which, already self-evident, was, thanks to their zeal, still farther confirmed by striking examples, and undeniable proofs. I am anxious to gratify this wish, which had been equally expressed by the National Institute. Men distinguished by their rank and talents have united themselves; forming a new society, the plan of which I now address to you. Of this society, the actual Members of the Central Committee of Vaccination, from their experience, and their devotion to the cause, ought to constitute a part. I now invite you to share their labours, and to second their exertions.

"It belongs more particularly to the Prefects, to extend through the departments the measures which the society has adopted; to the execution of which I am ready to dedicate all my attention.

"The advantages of vaccine inoculation are so obvious, and so easily attained, that the most certain method of making them known is, to enable every class of citizens to estimate their just value. It is, in fact, the peculiar nature of this discovery, to work its own way, and to propagate itself readily by the evidence of its utility, and of the benefits it confers. Being exempt from every kind of inconvenience, and, when once performed, not subjecting the patient to any expense, we are naturally prejudiced in favor of the practice; while the epidemic small-pox, so frequently recurring, and sparing, in the most populous neighbourhoods, when it rages in all its fury, those who have been inoculated with the cow-pock, offers a satisfactory demonstration of its efficacy to every observer.

"It is necessary to apply ourselves particularly to this practice, in order to give it new life; and to multiply, under the immediate inspection of the people, the opportunities of judging of its advantage. By recommending it to the institutors of lyceums, the managers of religious establishments, the proprietors of manufactories, and the governors of workhouses, who employ a great number of children, you will set our cities a salutary example.

"The children who are supported by the state, when vaccinated, vaccinated, and sent into the country, will also serve to make known the advantages of the new inoculation; unless, through the confidence of certain families in the skill of the faculty, or the zeal and good sense of some of the more intelligent inhabitants, it is already propagated there.

"The establishment of midwives, who are educated in the hospitals of Paris, in different parts of the country, the Juries of Medicine, and the sisters of charity, dispersed through the several communes, will prove still more the means of propagating the knowledge and practice of vaccination among the people. But above all, we must exert our utmost endeavours, to enlighten the public opinion; for which purpose, all the facts, and all the results of experiments, should be carefully collected. The most striking instances of preservation, observed during the epidemic small-pox, should be published; and if errors present themselves, or ignorance presume to make false allegations, the former should be carefully corrected, the latter speedily and forcibly refuted. A great number of results would contribute to fix the public opinion.

"The natural effect of propagating vaccination must be, to render the small-pox more and more rare. By keeping an annual register of the continual decrease of the number attacked with that disease, and of the proportion of its victims in our Bills of Mortality, we shall produce general conviction; and no cause will any longer retard the adoption of a practice, which is known to be the source of so great a benefit.

"This is the object we have in view; and, in order to attain it, we must dispute every inch of ground with the enemy, whom we wish to exterminate, by a wise combination of efforts; and by an union of measures which embrace every part of France. It appears to me necessary, that the example already set by some Prefects, should be followed by all; and that the course they pursue, is calculated to obtain uniform evidence upon every point, and unquestionable results from every quarter.

"This end will be accomplished, by establishing in every department a Committee of Vaccination, composed of the most intelligent medical practitioners, and associating with them citizens distinguished by their rank, fortune, and character. The ministers of the Gospel will be useful in these associations, on account of their influence. Many examples have taught us what services they may render on this occasion.

"We must commit to the disposal of every committee, in the towns where they are formed, one of the halls of the most frequented religious houses; with all the means necessary for constantly keeping up the practice of vaccination. In the towns of a sub-prefecture, committees of districts should be established, to correspond with the committee of the department; or we may supply the want of this measure, by appointing one or two of the most intelligent physicians, who shall be associated with the committee of the department.

"In order to extend vaccination through the country, one or two officers of health, in each canton, should be charged by the Prefect to vaccinate the poor gratuitously; or, if circumstances require it, the Prefect should appoint Professors of the art, who shall be directed to propagate inoculation through the country, at stated times. Such resources should be combined as situations admit, and opportunities offer; and those should be preferred, which promise the greatest advantage.

"The instructions and advice which may be deemed necessary, and the supplies of vaccine matter, shall be procured from the Committee of the Society; who shall answer every demand without delay.

"The Prefects shall address to me, every month, the result of the measures they adopt; and inform me of such medical practitioners, and zealous citizens, as shall distinguish themselves by their success, and their devotion to this cause; they shall also send to the society the observations they collect. I recommend to their attention the arrangement of the tables, a model of which is annexed. They will take care to send me two copies.

"Such are the means, Citizen Prefect, which I have thought it my duty to propose; the success of which, in my opinion, will be the more certain, in proportion as they approach to the plan adopted by the society. No object calls more loudly for your attention; it is one of the dearest interests of the state, and a certain mode of augmenting our population. By employing all the means in your power to enlighten the public opinion, by exciting the solicitude of families, and removing those obstacles, which the fear of a trifling expense too often opposes to the greatest benefit, you will manifest to all the citizens, even in the lowest ranks of society, the advantage of the new practice, which secures their preservation.

"On my part I will second your efforts, I will support you with all the power of Government; and, confident of your zeal, and that of the Society which devotes itself to the accomplishment of this good work in which we are all engaged, I doubt not but we shall, in a few years, annihilate the small-pox in France, as we have already annihilated the leprosy, and many other calamities of this kind; of which no traces are to be found, but in obscure or insulated cantons, or in the page of history.

"I have the honour to be, &c.

"CHAPTAL."

"*The Plan of the Society for the Extermination of the Small-pox in France, by Means of Vaccination.*

"The numerous experiments instituted in France, during the space of four years, prove, in the most incontestible manner, that the cow-pock is a security against the small-pox, by a process as certain in its effects, as it is mild and simple in its operation. Its success is established by more than a hundred thousand facts, verified by the Central Committee.

"During the four years that this Committee have pursued, with no less zeal than impartiality, the progress of vaccination, not a single fact has occurred that could shake the public confidence. It has been proved, that all which has been written to the contrary, has been the result of ignorance or of falsehood.

"We are now endeavouring to employ the means of diffusing the benefits of this salutary practice; and, by bringing it, as we hope, into general use, utterly to banish the small-pox. Such is the object of this new society forming in Paris, under the auspices of the Minister of the Interior; and which, already fortified by all the means Government can submit to its disposal, wishes to unite and concentrate every kind of knowledge, talent, reputation, and authority, in one point. The annexed ordinance of the Minister of the Interior, will explain the plan and organisation of the society.

"The extreme importance of this design, and the incalculable advantage which will result from its execution, leave no room to doubt, that every citizen, and every friend of humanity and of his country, will applaud the undertaking; and be eager to share our labours. Innumerable facts have demonstrated, that vaccination shields those who have submitted to it from the attack of the small-pox; and in the mean time, it is ascertained by proofs equally incontestible, that by multiplying it as oc-

 casion casion requires, we may banish that contagion from the most populous neighbourhood; and even from the precincts of a town or a city. Hospitals containing a thousand children, or more, have been thus preserved from an epidemic small-pox, by vaccinating those who had not undergone the disease. In some districts, where the epidemic small-pox had appeared, it was confined to, and extinguished in, the house where it first broke out, by vaccinating all the infants in the neighbourhood.

"To conclude, we have seen the infection, when spread over a large tract of country, arrested in its course at the gates of cities, where the inhabitants in general had been inoculated with the cow-pock. These memorable occurrences give us room to hope, that by propagating vaccination in every direction, and rendering the practice general, we may accomplish the extermination of the small-pox in France. This is the final object of our endeavours, the practicability of which is evident; and if we all cordially and strenuously unite in its execution, the French nation will set a great and glorious example to the world.

"*The Ordinance of the Minister of the Interior, concerning the Formation of a Central Society of Vaccination.*

"The Minister of the Interior, considering that the advantages of Vaccination have been sufficiently ascertained, by the numerous experiments made, or collected, by the Central Committee established at Paris, and by the National Institute; that this method, already practised with success in almost every part of France, only requires a regular and uniform mode of propagation, in order to obtain every degree of extension of which it is susceptible, has issued the following decree.

"1. There shall be at Paris, near the Minister of the Interior, a Central Society of Vaccination, of which the Minister shall be President.

"2. The Members of the Society are,

Delaplace, Chancellor of the Senate.

Lacepede, Grand Chancellor of the Legion of Honour.

Fontanes, President of the Legislative Corps.

Portalis, } Counsellors of State.

Fourcroy, } Counsellors of State.

Regnault (de S. Jean d'Angely) } Counsellors of State.

Bertholet, Senator.

La Rochefoucault Liancourt.

Corvisart, Physician of Government.

Coulomb, Secretary Gen. of the Minister of the Interior.

Thouret,

Thouret, Director of the School of Medicine.

J. J. Leroux, Professor of the School of Medicine.

Mongenot, Jadelot, } Physicians of the Hospital des Enfans.

Marin, Surgeon of the Lyceum.

Doussin Dubreuil, Guillotin, Salmade, Delaroche, } Doctors of Physic.

Parfait, } Member of the Central Bureau of Admission to the Hospitals.

Husson, Physician of the Hospital of Vaccination.

Hallé, Huzard, Tessier, Cuvier, Delambre, Parmentier, Pinel, Degerando, } Members of the National Institute.

Duquesnoy, Delessert, } Membs. of the Council Gen. of Hospitals.

Delasteyrie.

Coste, Physician of Invalids.

Barbier Neuville, } Chief of Division to the Minister of the Interior.

" 3. There shall be formed in the bosom of the Society, a Committee, consisting of sixteen Members, and the Secretary of the Society.

" 4. The Members of the Committee are Citizens Thouret, Corvisart, Pinel, Leroux, Hallé, Huzard, Guillotin, Salmade, Parfait, Delaroche, Marin, Jadelot, Delasteyrie, Doussin Dubreuil, Mongenot, and Husson.

" 5. The Secretary of the Society shall also be that of the Committee. The Minister appoints Cit. Husson to this situation.

" 6. The Prefects of the departments shall maintain a regular correspondence upon all subjects relative to vaccination, and to epidemic and epizootic small-pox; two copies of which shall be transmitted to the Minister; one for himself, and the other for the Society. The Prefects who shall be at Paris upon leave, may assist at the sittings of the Society.

" 7. Instructions shall be sent to the Prefects, with which they shall be requested to conform, as far as their situation will permit. The plans they have already adopted, for for propagating vaccination in their departments, shall be transmitted to the Minister, in order that, if necessary, and after the opinion of their Society has been taken, they may be sanctioned with his approbation.

"8. An annual report shall be made to the Society, at its public meeting, upon the labours undertaken in France for propagating vaccination; and upon the registers that shall be sent by the departments.

"9. Testimonials shall be granted, and rewards given, to those persons who have displayed the greatest zeal in propagating vaccination.

"This arret is dated the 14th of Germinal, and the 12th year of the Republic; and signed Chaptal, Minister of the Interior."

"*Regulations adopted by the Minister of the Interior, for the Committee of the Central Society of Vaccination.*

"1. The meetings of the Committee formed in the bosom of the Society, shall be held every Friday; and last from three to five hours.

"2. The Committee shall meet oftener, if necessary, on receiving notice from the President of the Society.

"3. The Committee shall correspond with the Prefects of Departments; and render an account every week to the Minister, and every month to the Society, of the facts relative to vaccination, which are contained in that correspondence.

"4. The President of the Committee is to be chosen by ballot, and by a majority of votes. He shall continue in office six months.

"5. The Secretary is to have the charge of all correspondence that is carried on in the name of the Committee, to take copies of the registers sent by the Prefects; to answer every thing relative to the science, and to send out supplies of cow-pock matter. He is to present, at every meeting of the Committee, a report of the labours of the week, to keep the minutes, to draw up the verbal process, and attend without delay to all letters and memoirs. He is to be the organ between the Committee and the Minister.

"6. The Secretary shall also answer all questions on the part of the administration of the Committees of the departments; but the answers on this subject shall be submitted to the General Meeting of the Society; and signed, if if possible, by the Minister, who is President of the Society.

"7. There shall be a General Meeting of the Society every month, in which the Secretary shall make a report upon the whole correspondence, the progress of the new practice, the improvements that may be expected, and the rewards that are to be conferred.

"I hereby certify, that this is a faithful copy of the original, entered in the verbal process of the meeting held by the Minister of the Interior, at Paris, on the 18th of Germinal, and the 12th year.

"Husson, Secretary.

"N.B. The correspondence, and applications for cow-pock matter, are to be addressed under cover to the Minister of the Interior, 'Au Cit. Husson, Docteur en Medicine, rue et école de Medicine, Paris."

